# The heart of a hibernator: EGFR and MAPK signaling in cardiac muscle during the hibernation of thirteen-lined ground squirrels, *Ictidomys tridecemlineatus*

**DOI:** 10.7717/peerj.7587

**Published:** 2019-09-05

**Authors:** Christine L. Childers, Shannon N. Tessier, Kenneth B. Storey

**Affiliations:** 1 Department of Biology, Carleton University, Ottawa, ON, Canada; 2 BioMEMS Resource Center & Center for Engineering in Medicine, Massachusetts General Hospital & Harvard Medical School, Charlestown, MA, USA; 3 Institute of Biochemistry, Department of Biology and Chemistry, Carleton University, Ottawa, ON, Canada

**Keywords:** Hibernation, MAPK, EGFR, *Ictidomys tridecemlineatus*, Cardiac hypertrophy, Heart

## Abstract

**Background:**

Thirteen-lined ground squirrels (*Ictidomys tridecemlineatus)* experience dramatic changes in physiological and molecular parameters during winter hibernation. Notably, these animals experience reduced blood circulation during torpor, which can put numerous stresses on their hearts. The present study evaluates the role played by the epidermal growth factor receptor (EGFR) in signal transduction during hibernation at low body temperature to evaluate signaling mechanisms. By investigating the regulation of intracellular mitogen activated protein kinase (MAPK) pathway responses, anti-apoptosis signals, downstream transcription factors, and heat shock proteins in cardiac muscle we aim to determine the correlation between upstream tyrosine phosphorylation events and downstream outcomes.

**Methods:**

Protein abundance of phosphorylated EGFR, MAPKs and downstream effector proteins were quantified using immunoblotting and Luminex^®^ multiplex assays.

**Results:**

Monitoring five time points over the torpor/arousal cycle, EGFR phosphorylation on T654, Y1068, Y1086 was found to increase significantly compared with euthermic control values particularly during the arousal process from torpor, whereas phosphorylation at Y1045 was reduced during torpor. Phosphorylation of intracellular MAPK targets (p-ERK 1/2, p-JNK, p-p38) also increased strongly during the early arousal stage with p-p38 levels also rising during prolonged torpor. However, of downstream MAPK effector kinases that were measured, only p-Elk-1 levels changed showing a decrease during interbout arousal (IA). Apoptosis markers revealed a strong reduction of the pro-apoptotic p-BAD protein during entrance into torpor that remained suppressed through torpor and IA. However, active caspase-9 protein rose strongly during IA. Levels of p-AKT were suppressed during the transition phases into and out of torpor. Of four heat shock proteins assessed, only HSP27 protein levels changed significantly (a 40% decrease) during torpor.

**Conclusion:**

We show evidence of EGFR phosphorylation correlating to activation of MAPK signaling and downstream p-ELK1 suppression during hibernation. We also demonstrate a reduction in p-BAD mediated pro-apoptotic signaling during hibernation with active caspase-9 protein levels increasing only during IA. *I. tridecemlineatus* has natural mechanisms of tissue protection during hibernation that is largely due to cellular regulation through phosphorylation-mediated signaling cascade. We identify a possible link between EGFR and MAPK signaling via p-ERK, p-p38, and p-JNK in the cardiac muscle of these hibernating mammals that correlates with an apparent reduction in caspase-9 apoptotic signaling. This reveals a piece of the mechanism behind how these mammals are resilient to cardiac stresses during hibernation that would otherwise be damaging.

## Introduction

Mammals that hibernate are an example of nature’s ability to adapt to environmental challenges. In the face of winter stresses, including low ambient temperatures and little or no access to food, hibernators have developed strategies to survive by greatly reducing their normal energy needs, entering a torpid state and letting body temperature (Tb) fall to near-ambient (often as low as 0–5 °C). Hibernation is characterized by cyclical periods of torpor (lasting several days to weeks) and brief periods of arousal (typically less than 1 day) when animals rewarm to euthermia (~37 °C). By reducing their metabolic rate during torpor to levels as low as 2–4% of euthermic values and relying on large reserves of body fat accumulated during summer feeding, hibernators can survive for many months without eating (Carey, Andrews & Martin, 2003; Wang & Lee, 2011). The strong metabolic depression that characterizes torpor is possible due to numerous adjustments to metabolism. For example, in ground squirrels these include (a) resetting the hypothalamic set-point to allow Tb to fall, (b) a reduction in breathing rate to ~2.5% of normal rate, (c) a decrease in heart rate from 350–400 beats/min in euthermia to 5–10 beats/min during torpor, (d) reduced organ perfusion to <10% of euthermia, and (e) suppression of high energy cellular processes such as transcription, translation, the cell cycle, ATP-dependent transmembrane pumps, etc. (Carey, Andrews & Martin, 2003; Geiser, 2004; Dark, 2005; Storey, 2010; Wu & Storey, 2012). All of this is reversed during arousal without damage to their tissues. Each tissue, however, can have their own specific adjustments that are needed in order to support animal survival during torpor, as demonstrated through proteome and transcriptome studies that reveal a drastic differences in response to torpor even between the two muscle tissues, skeletal and cardiac (Vermillion et al., 2015a, 2015b). Hibernator hearts in particular are known to respond with a reorganization of heart dynamics to increase the force of contraction in order to pump cold viscous blood through the squirrel body (Frerichs et al., 1994; Nakipova et al., 2007; Wang & Lee, 2011). Other hibernating squirrel species have been shown to use reversible cardiac hypertrophy to endure this stress, a mechanism that is commonly lethal in humans (Wickler, Hoyt & Van Breukelen, 1991; Hill & Olson, 2008; Nelson & Rourke, 2013; Shimizu & Minamino, 2016). In hibernating ground squirrels, however, cardiac hypertrophy is adaptive and reversible (Nelson & Rourke, 2013; Vermillion et al., 2015a). Hence, studies of the molecular changes that occur in hibernator hearts during hibernation could provide further insight into their natural adaptations and stress resistance. Many other studies have also indicated that the reduction in blood flow from hibernation is similar to cardiac ischemia and reperfusion, which these animals routinely endure over their torpor arousal cycles (Vermillion et al., 2015b; Yan et al., 2015). Thus, understanding the molecular signaling that occurs in the heart of these hibernators could provide insight into their ability to endure the fluctuations in oxygen delivery during torpor and arousal (Tessier & Storey, 2012; Luu et al., 2015; Zhang & Storey, 2016).

The epidermal growth factor receptor (EGFR) is a cell-surface site for the integration and transduction of signals from multiple types of stimuli (osmotic shock, oxidative radicals, peptide molecules and membrane depolarization, etc.) and is known to be an important signaling center in many pathological states such as diabetes, cancer, and cardiovascular dysfunction (Prenzel et al., 2001; Akhtar & Benter, 2013; Makki, Thiel & Miller, 2013). EGFR is sometimes a topic of debate in the literature with studies indicating both protective and adverse effects on heart cell health (Forrester et al., 2016). For example, the chronic administration of EGFR inhibitors for cancer treatment is thought to lead to heart failure (Barrick et al., 2008). Other studies suggest that EGFR is crucial for cardiac homeostasis, with many forms of this receptor tyrosine kinase being expressed in heart tissue (Fuller, Sivarajah & Sugden, 2008). Given the central importance of the heart in maintaining viability during hibernation, the present study uses this organ as a model to examine the role played by EGFR signaling in mediating cytoprotective responses in the hearts of thirteen-lined ground squirrels (*Ictidomys tridecemlineatus*) over their torpor/arousal cycle. Coordination of cytoprotective responses would surely be a necessity to achieve appropriate pro-survival actions (e.g., anti-apoptosis) and cell preservation processes (e.g., heat shock proteins and other chaperones) during cold torpor (Storey, 2010; Storey & Storey, 2011; Rouble et al., 2013).

Signaling through receptor tyrosine kinases at the plasma membrane typically promotes increased metabolism and cell growth. During hibernation, it is important that these receptors be poised to mediate a reduction in metabolic activity while retaining readiness to respond rapidly during arousal from torpor. Signal transduction cascades and the control that they exert over transcription factors have been shown to contribute to the hibernating phenotype by providing a mechanism to sense the external environment and alter gene expression programs accordingly (Storey, 2010; Tessier & Storey, 2012; Luu et al., 2015). EGFR is stimulated by several peptides including epidermal growth factor (EGF) and heparin-binding EGF. These ligands are synthesized as transmembrane precursors that must be cleaved by proteases to release mature ligands (Makki, Thiel & Miller, 2013). It has been previously demonstrated that EGFR agonists can induce cardiac hypertrophy and remodeling through the activation of metalloproteinases that cleave and release EGFR ligands (Asakura et al., 2002). Ligand binding to EGFR triggers auto phosphorylation of the receptor at specific tyrosine residues that leads onward to phosphorylation-dependent interactions with many adaptor proteins. These interactions include outcomes such as activating stress signaling pathways and receptor internalization mechanisms (Seet et al., 2006). For example, the docking of several SH2 domain-containing adaptor proteins to EGFR activates stress signaling pathways such as the mitogen activated protein kinase (MAPK) pathway.

MAPK signaling is known to play crucial roles in regulating cell responses to diverse stresses (Seger & Krebs, 1995; Wada et al., 2001; Gehart et al., 2010; Wortzel & Seger, 2011) and several studies have documented MAPK-mediated actions during hibernation at low Tb values (MacDonald & Storey, 2005; Zhu et al., 2005; Tessier et al., 2017). MAPKs relay stress signals from external stimuli through phosphorylation cascades that ultimately affect an array of downstream targets (Seger & Krebs, 1995; Cowan & Storey, 2003; Lawrence et al., 2008; Booy, Henson & Gibson, 2011). Due to this, hibernating mammals have become an important platform with which to study natural mechanisms of MAPK signaling that may be applied to the treatment/prevention of diseases involving tissue degeneration and pathological stress responses, including conditions characterized by cardiomyopathy (Storey, 2010; Storey & Storey, 2010).To elucidate the underlying molecular patterns that mediate cardiac adaptation and the adjustments to the cooling of hibernator hearts during natural hibernation, this study assessed the regulation of EGFR signaling and downstream MAPK pathways. We measured the relative protein expression of downstream effectors of such responses, as well as the possible signal transducers that may be responsible for relaying the appropriate molecular messages toward the promotion of stress-tolerance, over the course of the torpor–arousal cycle in hearts of *I. tridecemlineatus*. The hearts of hibernators must naturally adjust to low temperatures and to the reduced perfusion that would be considered ischemic during euthermia or in non-hibernating species (Frerichs et al., 1994). Hibernator hearts strategically decease cellular energy demands to match ATP production and continue to beat at temperatures far below those that cause hypothermic cardiac arrest in most other mammals (Liu, Wang & Belke, 1993; Wang et al., 2002). Therefore, anti-apoptosis signaling is predicted to be a crucial factor in maintaining heart viability during hibernation. Indeed, a previous study found that the STAT1,3, and 5 transcription factors that can mediate apoptosis signaling are increased during torpor in the thirteen-lined ground squirrel muscle (Logan et al., 2016). Since STAT1 is involved with pro-apoptotic functions whereas STAT3 and 5 are involved with pro-survival gene expression and cardio protection, the state of apoptosis signaling is of interest in this hibernator model (Barry et al., 2007; Boengler et al., 2008; Cambi et al., 2012). Indeed, the tissue-preservation mechanisms in hibernators may be useful in the development of strategies to lengthen the viability of vital organs, such as hearts, that are removed for transplantation.

## Materials and Methods

### Animal experiments

Animal experiments were performed as described previously in Rouble et al. (2013) and were carried out by the laboratory of Dr. J.M. Hallenbeck at the Animal Hibernation Facility, National Institute of Neurological Disorders and Stroke (NIH, Bethesda, MD, USA). NINDS Animal Care and Use Committee (ACUC) approved animal housing and experimental procedures were followed (protocol number ASP 1223-05). Briefly, thirteen-lined ground squirrels (*I. tridecemlineatus*), weighing 150–300 g, were wild-captured by a United States Department of Agriculture-licensed trapper (TLS Research, Bloomingdale, IL, USA) and transported to the NIH. Animal housing and experimental procedures followed the guidelines set by the NINDS ACUC. Animals were individually housed in shoebox cages at 21 °C and fitted with a sensor chip (IPTT-300; Bio Medic Data Systems, Seaford, DE, USA) injected subcutaneously while anesthetized with 5% isofluorane. Animals were fed standard rodent diet and water ad libitum until they gained sufficient lipid stores to enter hibernation. To enable a natural transition into torpor, animals were transferred to an environmental chamber at ∼5 °C in constant darkness. Tb, time and respiration rate were monitored and used to determine sampling points. All animals had been through a series of torpor–arousal bouts prior to sampling. Tissue samples were shipped to Carleton University on dry ice. Tissues were stored at −80 °C until use. EC designates “euthermic control” animals, these euthermic squirrels had a stable Tb (∼37 °C) in the 5 °C cold room, could enter torpor but had not done so in the past 72 h; EN designates “entrance” into hibernation with a falling Tb of 18–31 °C; LT indicates “long term” torpor with a stable Tb at 5–8 °C for at least 5 days; EA designates “early arousal” from torpor with a rising Tb of 9–12 °C, and IA designates the “interbout arousal” where animals were fully aroused for >18 h with Tb restored to 37 °C (McMullen & Hallenbeck, 2010).

### Luminex^®^ assays

Luminex^®^ assay panels were purchased from EMD Millipore and were used according to the manufacturer’s instructions. The assays employed in this study were used to measure EGFR autophosphorylation at specific tyrosine residues (8-plex EGFR profiler Magnetic Bead Kit, Cat#48-613MAG) in heart muscle of thirteen-lined ground squirrels at specific time points. The multiplex EGFR profiler kit was used to determine the relative phosphorylation levels of EGFR in response to hibernation at the following residues: pT654, pT669, pY845, PY1045, pS1047, pY1068, and pY1086. The 10-Plex MAPK/SAPK Signaling Panel (Cat#48-660MAG) was used to determine phosphorylated protein levels of specific MAPK/SAPK signaling factors. Targets evaluated include MEK1 (Ser221), ERK/MAP Kinase 1/2 (Thr185/Tyr187), p38 (Thr180/Tyr182), JNK (Thr183/Tyr185). The 7-plex early apoptosis magnetic bead panel was used to determine active apoptosis components that include p-JNK (Thr183/Tyr185), p-BAD (Ser112), p-Akt (pS473), and active caspase 9 (Asp315). The 5-plex heat shock protein magnetic bead panel (Cat#48-615MAG) was used to measure the total protein levels of HSP27, p-HSP27(S78), p-HSP27(S78/s82), HSP60, HSP70, and HSP90alpha.

Positive and negative controls provided by the manufacturer were used with each assay panel and were prepared according to the manufacturer’s instructions. Extracts of frozen tissue samples were prepared as per manufacturer’s instructions (Biggar et al., 2015). Briefly, ∼50 mg samples were weighed and homogenized in lysis buffer with two mM phenylmethylsulfonyl fluoride added. Samples were then frozen at −80 °C and thawed at room temperature twice before they were centrifuged at 4,000×*g* for 4 min and supernatants were collected as total soluble protein lysates. Protein concentration was determined using the Bradford assay with the Bio-Rad prepared reagent and then further diluted to an appropriate concentration using assay buffer. The protocol for multiplex analysis followed manufacturer’s directions as described in Biggar et al. (2015). Measurements were taken immediately after the assay was finished using a Luminex 100^®^ instrument with xPonent software (Luminex^®^ Corporation, Austin, TX, USA).

### Total protein extraction and immunoblotting

Total protein extraction from tissue samples, SDS-polyacrylamide gel electrophoresis, protein transfer to PVDF membranes, and immunoblotting was performed as described previously (Tessier et al., 2017). Membranes were blocked using 2.5% skimmed milk in TBST for 20 min and were probed with specific primary antibodies for mammalian CREB-1 phosphorylated at Ser133 (Cat#7978-R; Santa Cruz Biotechnology Inc., Dallas, TX, USA) and Elk-1 phosphorylated at Ser383 (Cat#9181; Cell Signaling Technology, Danvers, MA, USA) (both 1:1,000 v:v dilution in TBST) at 4 °C overnight. Membranes were then probed with secondary anti-rabbit IgG HRP-linked antibody (1:4,000 v/v dilution in TBST) for 20 min at room temperature and developed using enhanced chemiluminescence.

### Quantification and statistical analysis

Bead-based assays used the net median fluorescence intensity of a population of measurements (with a minimum bead count of 50 with subtraction of background wells) to determine relative protein levels. Immunoblot protein bands were visualized using a ChemiGenius Bio Imaging System (Syngene, Frederick, MD, USA) and quantified by densitometric analysis with the associated GeneTools Software (Syngene, Frederick, MD, USA). Immunoblot band density was standardized against the summed intensity of a group of Coomassie-stained protein bands from the same sample lane, the latter chosen based on their lack of variation between experimental conditions and because they were not located close to the protein band of interest. This method of standardization was employed due to the lack of change in the expression of most proteins found in hibernator tissues across the torpor–arousal cycle. Data are expressed as mean ± SEM (*N* = 3–4 independent samples from different animals) and were analyzed using a one-way ANOVA and a post hoc Tukey test (SigmaPlot 12 software). Differences between means were considered significant at *p* < 0.05.

## Results

The responses of EGFR and multiple signaling molecules, transcription factors and chaperone proteins over the course of the torpor–arousal cycle were assessed in heart tissue from thirteen-lined ground squirrels at five stages. These were: euthermic animals in the 5 °C cold room (EC), entry into hibernation with a falling Tb of 18–31 °C (EN), LT torpor with a stable Tb at 5–8 °C for at least 5 days (LT), EA from torpor with a rising Tb of 9–12 °C (EA), and fully aroused for >18 h with Tb restored to 37 °C (IA) (McMullen & Hallenbeck, 2010).

### Epidermal growth factor receptor phosphorylation in heart over the torpor–arousal cycle

**Figure 1:**
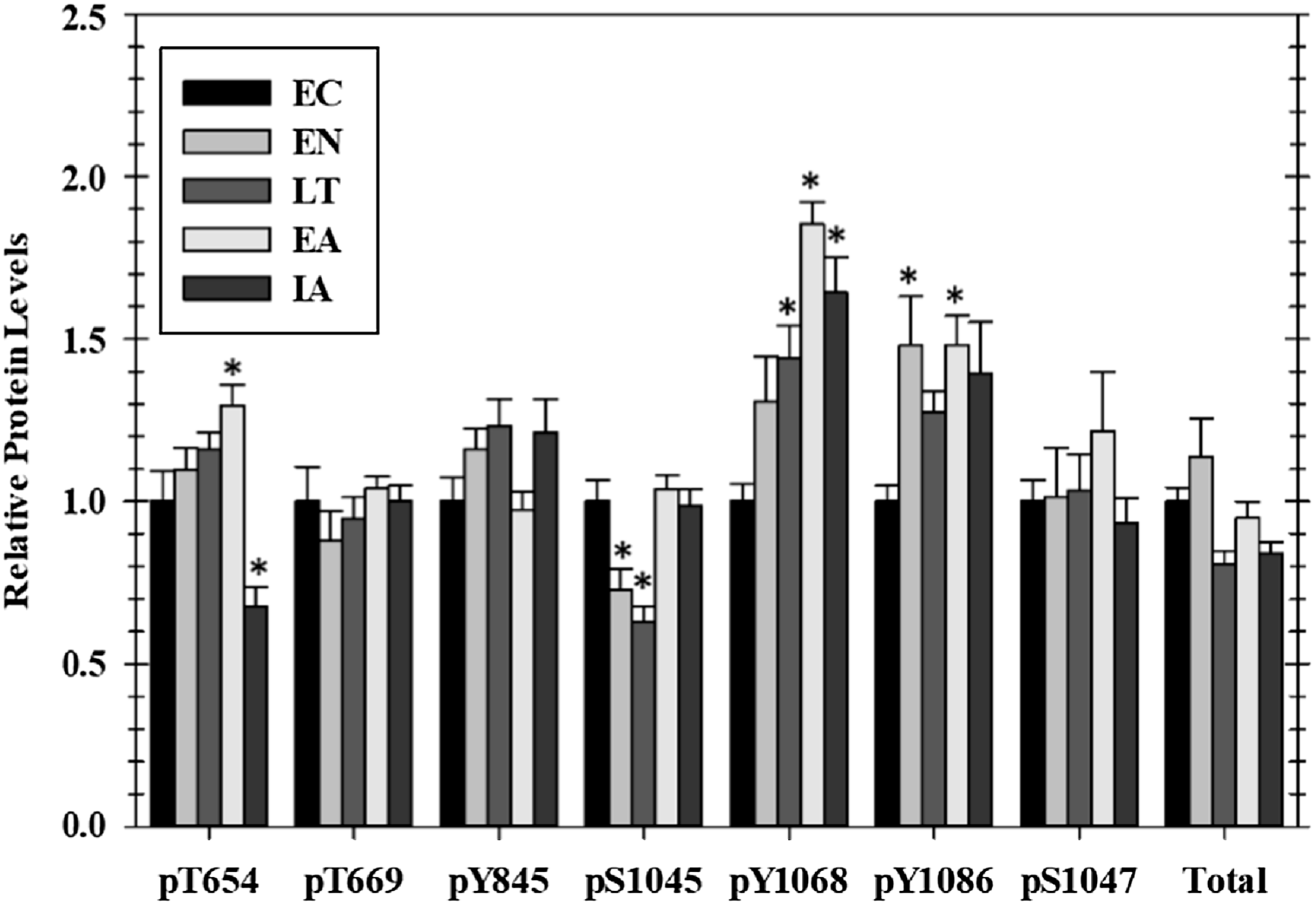
Relative phosphorylation status of EGFR in cardiac muscle of *I. tridecemlineatus* over the torpor–arousal cycle. Data for seven phosphorylation sites on EGFR were acquired as well as for total EGFR protein. Histograms showing relative protein levels for bead-based assays were produced using the net MFI as measured by Luminex^®^. Data are means ± S.E.M. *N* = 3–4 independent protein isolations from different animals. Asterisks indicate values that are significantly different from the corresponding EC value, *p* < 0.05.

Of the seven EGFR phosphorylation sites that were assessed, four showed significant changes as compared with EC controls over the course of the torpor arousal cycle: pT654, pY1045, pY1068, and pY1086. Phosphorylation on T654 showed an increasing trend throughout hibernation and peaked at 1.3-fold higher in EA, compared with EC before dropping by ∼50% after animals were fully aroused back to euthermia (Fig. 1). At Y1045 phosphorylation was decreased 44.3% and 50.4% during EN and LT time points but levels returned to EC levels during EA and IA. Phosphorylation on Y1068 increased incrementally to 1.4-fold higher during LT and peaked at 1.9-fold higher in EA before falling slightly back to 1.6-fold higher than EC during IA (Fig. 1). Phosphorylation of Y1086 was generally elevated over EC at all experimental points with significant increases of 1.48-fold seen in both the two transition phases, EN and EA (Fig. 1). However, the relative phosphorylation state of pT669, pY845, and pS1047 as well as total EGFR protein content did not change over the torpor–arousal cycle.

### Regulation of MAPK signaling cascades in cardiac muscle over the torpor arousal cycle

**Figure 2:**
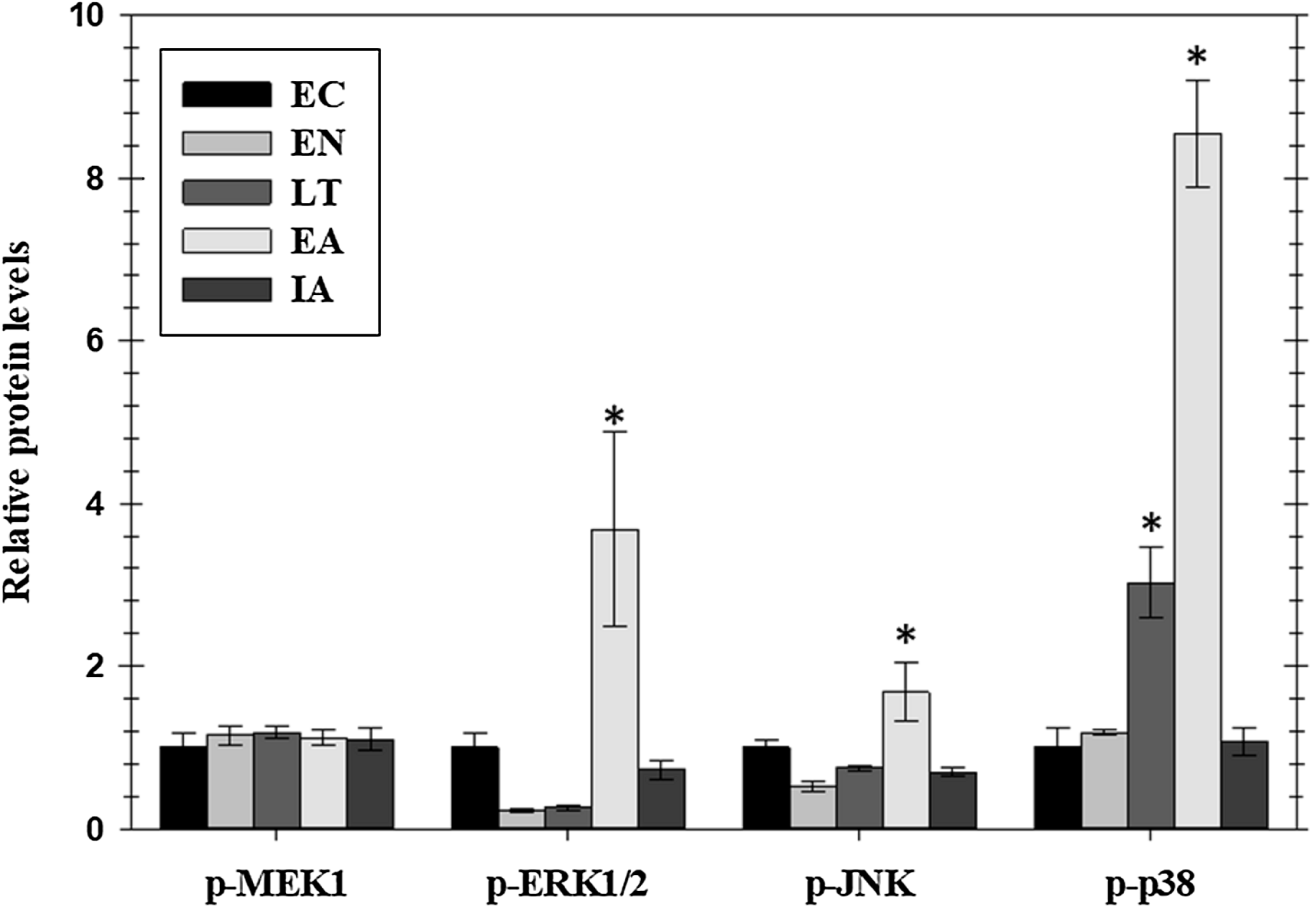
Relative phosphorylation status of the MAPK signaling pathway kinases MEK1 (Ser222), ERK1/2 (Thr185/Tyr187), JNK (Thr183/Tyr185), and p38 Thr180/Tyr182 in cardiac muscle of *I. tridecemlineatus* over the torpor–arousal cycle. Histograms showing relative protein levels for bead-based assays were determined using the net MFI as measured by Luminex^®^. Data are means ± S.E.M. *N* = 3–4 independent protein isolations from different animals. Asterisks indicate values that are significantly different from the corresponding EC value, *p* < 0.05.

Phosphorylated protein levels of several members of the MAPK-signaling pathway, representing the three main MAPK groups (ERK, JNK, and p38), also changed significantly in ground squirrel heart over the course of a hibernation bout (Fig. 2). Phosphorylation is known to activate these proteins and all three consistently showed increased phosphorylation during the EA period from torpor. Levels of p-ERK1/2 increased significantly by 3.7-fold in EA as compared to EC whereas p-JNK increased by 1.7-fold. Levels of p-p38 were significantly higher than EC during LT (3.0-fold) and rose further during EA (8.4-fold). However, phosphorylation of MEK1, an upstream kinase that regulates ERK1/2, did not change over the torpor/arousal cycle.

### Regulation of MAPK effector kinases and transcription factors in cardiac muscle during hibernation

**Figure 3:**
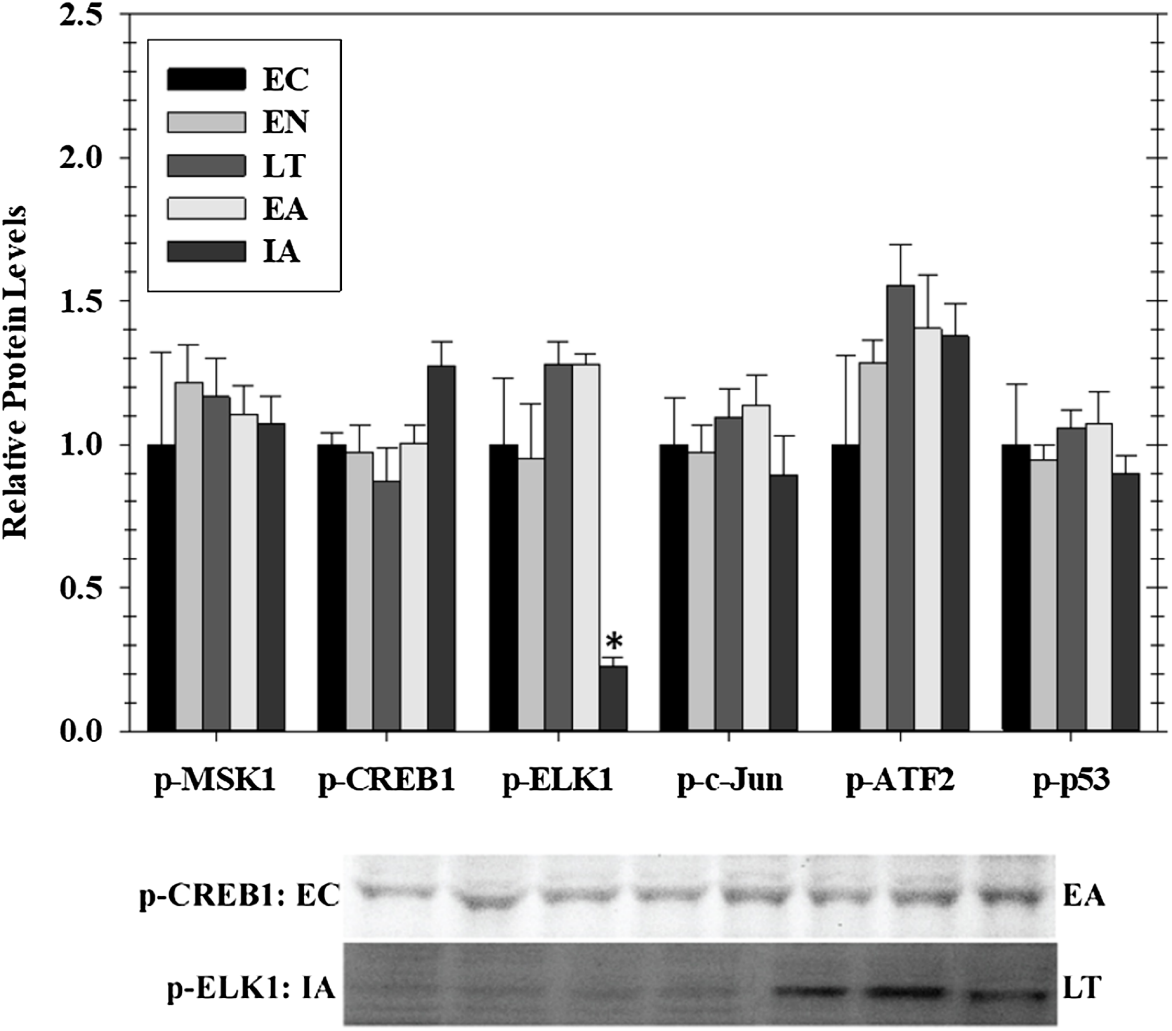
Relative phosphorylation status of the effector kinase p-MSK1 (Ser212) and transcription factors p-CREB1 (Ser133), p-ELK1 (Ser383), p-c-Jun (Ser73), p-ATF2 (Thr71), p-p53 (Ser15) in cardiac muscle of *I. tridecemlineatus* over the torpor–arousal cycle. Histograms show relative protein levels determined using the net MFI for bead-based assays (p-c-Jun Ser73, p-ATF2 Thr71, p-p53 Ser15) or from relative band densities for immunoblot analysis (p-CREB1 Ser133, p-ELK1 Ser383). Representative immunoblots are shown for selected time-points for p-CREB1 (EC vs EA) and p-ELK1 (IA vs LT). Data are means ± S.E.M. *N* = 3–4 independent protein isolations from different animals. Asterisks indicate values that are significantly different from the corresponding EC value, *p* < 0.05.

Transcriptional activity is regulated by posttranslational modifications, such as protein phosphorylation, at several distinct sites on transcription factors. MAPKs are a class of protein kinases that can phosphorylate transcription factors. Known transcription factors targets of MAPKs (and the sites that they phosphorylate) include p-CREB (Ser133), p-ELK1 (Ser 383), p-c-Jun (Ser73), p-ATF2 (Thr 71), and p-p53 (Ser15). These are either directly phosphorylated by MAPKs or by MAPK phosphorylation of an intermediate kinases (e.g., Msk1 Ser212). Hence, we assessed phosphorylated protein levels of five well-known stress-activated transcription factors in cardiac muscle over the course of hibernation using luminex and immunoblotting (Fig. 3). Overall, we detected no significant change in the phosphorylation state of these factors in heart over torpor–arousal. The only exception was a significant decrease in p-Elk1 (S383) levels during IA (decreased by 77%) when compared to EC.

### Expression of anti-apoptotic signals in cardiac muscle over the torpor arousal cycle

**Figure 4:**
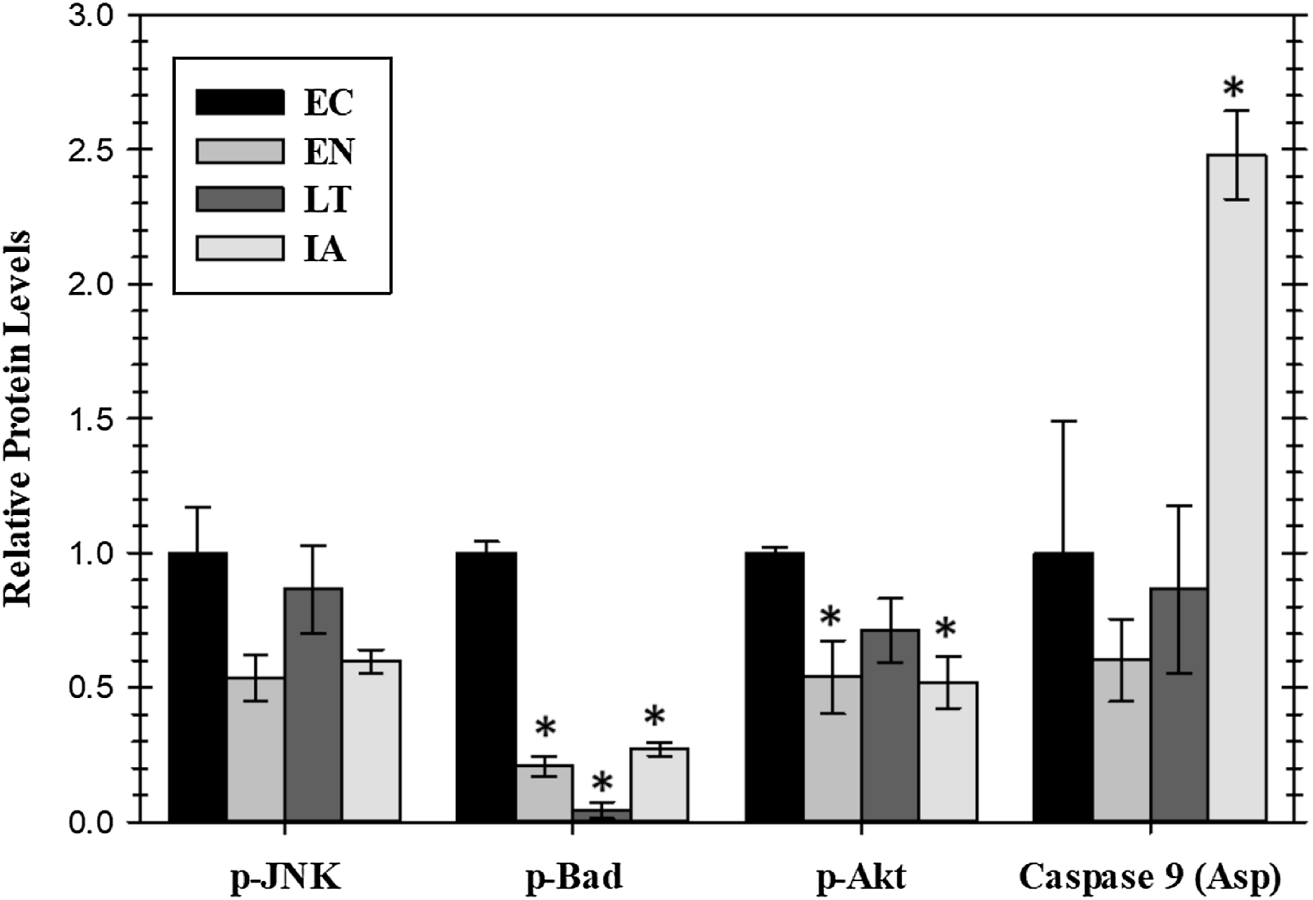
Relative protein levels of p-JNK (Thr183/Tyr185) and p-Akt (pS473) protein kinases and downstream pro-apoptotic proteins, p-BAD (Ser112) and active caspase 9 (Asp315) in the cardiac muscle of *I. tridecemlineatus* over the torpor–arousal cycle. Histograms showing relative protein levels were determined using the net MFI for bead-based. Data are means ± S.E.M. *N* = 3–4 independent protein isolations from different animals. Asterisks indicate values that are significantly different from the corresponding EC value, *p* < 0.05.

Extracellular signaling through EGFR can also lead to the regulation of anti-apoptotic signals mediated through the action of the AKT or JNK protein kinases or by the proteins BAD (Bcl-2-associated death promoter) and caspase-9. Relative levels of these four proteins were assessed for four stages of the torpor–arousal cycle (EC, EN, LT, and IA) (Fig. 4). A crucial finding was the very strong suppression of the pro-apoptotic protein BAD with values decreasing significantly during EN, LT, and IA to just 21%, 4.4%, and 27%, respectively, of the EC value. Oppositely, caspase-9 protein levels increased strongly by 2.5-fold during IA. Both p-JNK and p-Akt protein levels showed an oscillating pattern with p-Akt protein levels significantly reduced by about 50% in both EN and IA, as compared to EC (Fig. 4). However, data for JNK protein levels did not show significant changes.

### Expression of heat shock proteins in cardiac muscle over the torpor arousal cycle

**Figure 5:**
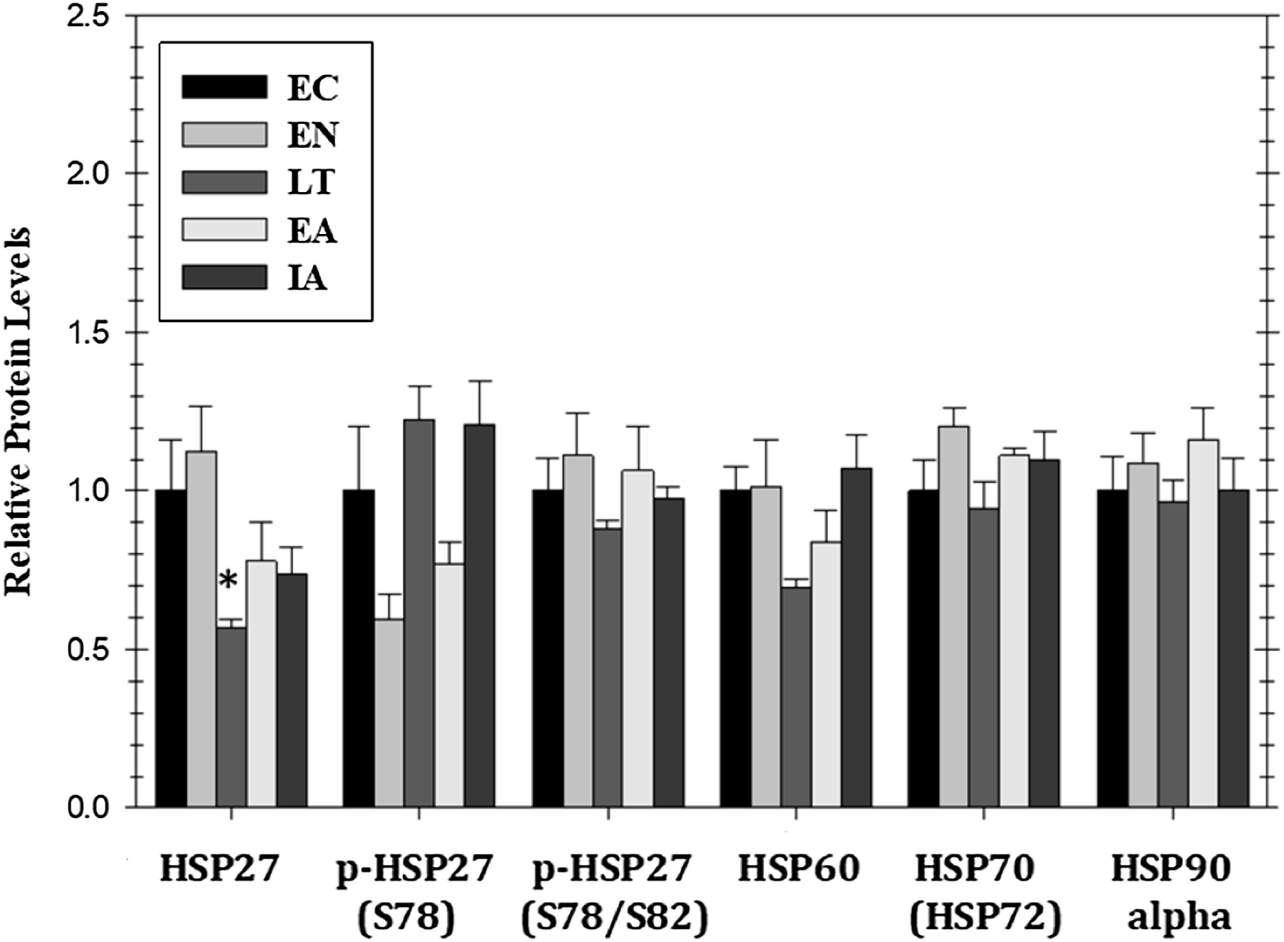
Relative protein expression of selected heat shock proteins and their phosphorylated forms in cardiac muscle of *I. tridecemlineatus* over the torpor–arousal cycle: HSP27, p-HSP27 Ser78, p-HSP27 Ser78/Ser82, HSP60, HSP70 (HSP72), and HSP90alpha. Histograms showing relative protein levels for bead-based assays were determined using the net MFI as measured by Luminex^®^. Data are means ± S.E.M. *N* = 3–4 independent protein isolations from different animals. Asterisks indicate values that are significantly different from the corresponding EC value, *p* < 0.05.

Mitogen activated protein kinase action can also affect the expression of heat shock proteins that are pivotal to the stress response. Figure 5 shows responses by four HSPs (HSP27, HSP60, HSP70/72, HSP90alpha) as well as relative phospho-HSP27 content over five stages of the torpor–arousal cycle. Protein levels of HSP27 decreased by 44% during LT as compared to EC but the relative phosphorylation of HSP27 at Ser78 or Ser78/Ser82 was unchanged relative to EC. Protein levels of HSP90alpha, HSP70(HSP72), and HSP60 did not change significantly over any of the stages of hibernation (Fig. 5).

## Discussion

The present study was designed to further our understanding of the molecular regulation of heart survival during hibernation at low Tb in thirteen-lined ground squirrels. EGFR signaling is known as a molecular integration site in pathological states such as cardiovascular dysfunction and cancer and the inhibition of this receptor has become a common treatment in the management of the latter (Barrick et al., 2008). This treatment option has become problematic, however, since the chronic inhibition of EGFR may lead to cardiac dysregulation (Barrick et al., 2008). The protected cardiac function seen in hibernating ground squirrels therefore provides a natural model system that can be compared to processes that maintain cardiomyocyte viability (Zhang, Aguilar & Storey, 2016). It is of interest to highlight the regulatory responses from cell surface receptors down to transcription factors and heat shock proteins in order to understand the biochemical pathways that are involved in the regulation of cardiac muscle durability during hibernation. The fast activation and stabilization of pro-survival pathways through the addition of posttranslational modifications may be crucial to the maintenance of the prolonged hypometabolic state of hibernation that includes a global suppression of transcription and translation (Storey, 2010). Here, we hypothesized that a cascade of phosphorylation changes emanating from signals received at the cell surface by EGFR would occur to promote anti-apoptotic pathways through MAPK signaling kinases and transcription factors that are downstream of EGFR in cardiac muscle. Accordingly, the B-cell lymphoma 2 (Bcl-2)-associated death promotor was significantly reduced during hibernation indicating a reduction in apoptotic signals while the animals are in torpor. This was balanced by a comparable reduction in proliferation signals from p-AKT demonstrating a coordinated system that maintains energy balance even as overall metabolic rate is suppressed to very low values (Fig. 4).

The activation of EGFR generates tyrosine-phosphorylated recognition motifs for the binding of signaling proteins containing SH2 domains. The data for EGFR phosphorylation (Fig. 1) demonstrates that the receptor is differentially phosphorylated on four sites at selected stages of the torpor–arousal cycle and thus may actively trigger downstream signaling to alter cardiac metabolism. Phosphorylation increased sequentially on Y1068 becoming statistically increased in late torpor and remaining high through early and IA, whereas phosphorylation on Y1086 increased during EN and EA time points (Fig. 1). Phosphorylation on Y1068 and Y1086 promotes the docking of Grb2 protein which can lead to the activation of Ras and its downstream kinase ERK1/2 (Lowenstein et al., 1992; Buday et al., 1994; Bogdan & Klämbt, 2001). Interestingly, we found that phosphorylated ERK1/2 levels were increased during EA. Taken together, these data suggest that EGFR signaling at these sites could be activating downstream ERK1/2 signaling during the early stages of arousal from torpor (Figs. 1 and 2). ERK1/2 has previously been implicated in a heart hypertrophic response therefore the EGFR signaling in EA correlating to ERK1/2 activation could be a mechanism for stimulating hypertrophy during periods of arousal in hibernation (Bueno & Molkentin, 2002). Oppositely, phosphorylation of EGFR at Y1045 was reduced during EN into torpor and remains significantly lowered in the late torpor time point before returning to control levels during IA. Phosphorylation of Y1045 creates the docking site for Cbl, an adaptor protein thought to be responsible for directing multi-ubiquitinylation and downregulation of EGFR (Thien & Langdon, 2001; Bogdan & Klämbt, 2001). The suppression of tyrosine phosphorylation at this site during torpor could be a mechanism to spare EGFR from becoming internalized and potentially degraded, thereby helping to maintain functional EGFR throughout the metabolic rate depression of torpor. Phosphorylation also increased on T654 residues in EA and then dropped in IA compared to ECs (Fig. 1). Phosphorylation of EGFR at T654 is a PKC target site that is thought to negatively regulate EGFR activity without degradation when it is phosphorylated (Bao et al., 2000). This could be crucial during the initial arousal during torpor when new protein synthesis is limited, and therefore an inhibition of EGFR function without destruction of the protein could be an important layer of control to suppress EGFR signaling as the animal warms. The large drop in T654 phosphorylation between EA and full IA could represent an off/on signal brought about by a dephosphorylation event that helps to reinstate downstream signaling mediated by EGFR once the animal returns to euthermia. Overall, these subtle shifts in phosphorylation patterns during the different stages of hibernation indicate a mechanism for making changes in downstream signaling via the EGFR over the various stages of hibernation. The early phosphorylation at Y1068 and Y1086 indicates the potential mechanism by which EGFR signaling can trigger downstream MAPK regulation through the ERK 1/2 pathway.

Mitogen activated protein kinase signaling pathways are known to be crucial to the regulation of the cellular stress response and have been previously implicated in hibernation and torpor (Yue et al., 2000; MacDonald & Storey, 2005; Abnous, Dieni & Storey, 2012; Rouble, Tessier & Storey, 2014; Tessier et al., 2017). Interestingly, MAPKs are known to have a broad range of functions and have been identified as participating in opposing functions depending on the strength and nature of the stimuli (Lawrence et al., 2008; Gehart et al., 2010). MAPKs can ultimately lead to the activating of transcription factors that are integrated in the stress response of cardiac tissue (Heineke & Molkentin, 2006). Our analysis of the MAPK signaling pathway in thirteen-lined ground squirrel heart indicated that there are major changes in phosphorylated protein levels of MAPKs across the torpor–arousal cycle that follow upstream EGFR signaling (Fig. 2). The phosphorylation states of ERK1/2, JNK, and p38 all increased strongly during the EA period, following the pattern of EGFR Y1086 phosphorylation, with phospho-p38 levels also rising earlier during late torpor paralleling the enhanced phosphorylation of EGFR Y1068 (Figs. 1 and 2). Previous work has suggested that ERK has a protective role in the heart whereas p38 and JNK mediate apoptosis pathways and the balance of these pathways can determine cell fate under stress (Yue et al., 2000). The increase in cardiac p-JNK is similar to data for skeletal muscle of thirteen-lined ground squirrels that also showed increased phosphorylation during late torpor and EA (Tessier et al., 2017). However, skeletal muscle did not show an increase in p-p38 or ERK1/2 (Tessier et al., 2017) and this suggests that the protective mechanisms at play in cardiac muscle may be more complex than in skeletal muscle. This is not surprising since the heart must retain its ability to beat during torpor whereas skeletal muscle may be fully dormant. Interestingly, whereas both brown adipose tissue and skeletal muscle can contribute to thermogenesis during arousal from torpor, brown adipose does so immediately whereas skeletal muscle shivering only starts once Tb warms to about 15 °C, indicating a delay in reawakening skeletal muscle. Hence, these results for ground squirrel heart indicate that p-ERK1/2, p-JNK, and p-p38 may act together in a tissue specific manner to regulate the functions of selected transcription factors or other downstream targets in response to EGFR signaling in order to help coordinate metabolic “reawakening” during arousal from torpor.

To further assess the potential effects of EGFR induced signaling, this study investigated the downstream phosphorylation of some transcription factors, anti-apoptotic signals, and HSPs that are known to be involved in ischemia protection and hypertrophy. The phosphorylation of MSK1 (a downstream kinase) and selected transcription factors that are downstream of MAPK signaling (CREB1, ELK1, c-Jun, ATF2, and p53) were assessed in heart over the five timepoints across the hibernation cycle. Interestingly, among these targets there was a change in the phosphorylation state of only one, ELK1, that was strongly suppressed during IA (Fig. 3). ELK1 is an ETS-domain transcription factor that regulates core gene regulation machinery including transcriptional complex components like c-Fos (Boros et al., 2009; Besnard et al., 2011). ELK1 is strongly activated by phosphorylation (measured on ELK1 at S383) and is a well-known target of ERK, JNK, and p38 MAPKs (Cruzalegui, Cano & Treisman, 1999; Aplin et al., 2001). Interestingly, p-ELK1 levels decreased during IA while upstream activation of ERK1/2, JNK, and p38 was not significantly different from EC animals. This is opposite to the results reported for skeletal muscle that found a significant increase in p-ELK1 in late torpor and through to IA along with the upstream activation of JNK (Tessier et al., 2017). The increase in upstream kinase activity in the heart is perhaps due to the maintained activity of the heart, while skeletal muscle remains dormant during torpor (Vermillion et al., 2015a; Tessier et al., 2017). Furthermore, this activity may be used to maintain control over various metabolic processes while the animals are torpid but may be counteracted by another signal when the animals start to warm up during arousal. Since ELK1 is responsible for coordinating the regulation of the basal transcription machinery, a reduction of its binding during IA could be a mechanism of suppressing global transcription during the brief warming of the animal so that gene expression is concentrated only on specific targets that need attention before animals sink into another torpor bout (Boros et al., 2009).

Akt is typically a downstream kinase responding to insulin and regulates various anabolic processes such as cellular growth. It has been shown to play an important role in other ground squirrel tissues during hibernation and is required for heart growth (Heineke & Molkentin, 2006; McMullen & Hallenbeck, 2010). In cardiac muscle, p-Akt protein levels were reduced during EN into hibernation and in IA as compared to EC (Fig. 4). This signaling may assist in blocking cell growth signals during periods when survival and apoptotic signals are in flux while allowing signaling to continue once a homeostasis is achieved such as during IA when ERK1/2 signals are also activated. The phosphorylation state of anti-apoptotic signals was assessed previously in cardiac muscle of EC versus LT squirrels. Rouble et al. (2013) analyzed the Bcl-2 family of proteins, known to regulate apoptosis at the mitochondrial level. Anti-apoptotic proteins were found to be differentially phosphorylated in squirrel heart during late torpor (implicating an action to promote cell survival) with the exception of Bcl-xL and Mcl-1 (Rouble et al., 2013). The present study analyzed protein levels of BAD, a pro-apoptotic member of the Bcl-2 family that promotes cell death, showing that this protein was strongly suppressed during torpor. BAD is a downstream target of both the MAPK and Akt pathways. Both these upstream protein kinase pathways need to be inhibited for BAD to become significantly dephosphorylated and thus activate its pro-apoptotic functions. Since p-BAD protein levels are greatly reduced and EGFR/MAPK signals are maintained, the slight reduction in p-AKT levels is likely not enough to allow apoptosis and is likely a result of lower growth signaling associated with the nonfeeding state of torpor (She et al., 2005). In accordance with the suppression of pro-apoptotic p-BAD during torpor, it was notable that active caspase nine levels only increased during IA when its allosteric inhibitor, Akt, was also reduced (Fig. 4). Hence, apoptosis to clear damaged cells may occur but appears to be restricted to times the animal has rewarmed to euthermia.

Heat shock proteins represent essential targets of MAPK pathways; for example, HSP27 is a terminal substrate of the p38 pathway and is known to be dephosphorylated during ischemia (Armstrong, Delacey & Ganote, 1999). In ischemic preconditioning studies, HSP27 was dephosphorylated at a slower rate after preconditioning which suggests that the phosphorylation of this HSP may be a mechanism of protection during acute cardiac stress events (Armstrong, Delacey & Ganote, 1999). The present study found a similar response since although HSP27 protein levels were reduced in LT as compared to EC the phosphorylation state of HSP27 remained constant across the time course potentially maintaining resistance to lower oxygen perfusion (Fig. 5). Interestingly, p-p38 levels were increased in LT and EA with no matching increase in p-HSP27. This may indicate that HSP27 has a negative regulator that acts against this p38 activation, however, more experiments are needed to investigate this mechanism. The phosphorylation of HSP27 facilitates actin polymerization and the formation of stress fibers. A reduction HSP27 protein as squirrels cool down and modify their cellular signaling may reduce the formation of such stress fibers during LT (Landry & Huot, 1995). A possible result of a reduction in stress fiber formation may be the inhibition of mitosis, since it is necessary to conserve energy throughout torpor by reducing non-essential anabolic activities. However, the lack of change in other HSPs over the torpor–arousal cycle suggests that, in general, the conditions of hibernation do not constitute an external stress on ground squirrel hearts which fits with the idea that changes to heart physiology during hibernation is a regulated and coordinated physiological state.

## Conclusions

*Ictidomys tridecemlineatus* has natural mechanisms of transcriptional regulation during hibernation through changes in the phosphorylation of receptor tyrosine kinases such as EGFR and their downstream signaling pathways. The current data suggest that the phosphorylation of MAPKs, such as ERK1/2, act as potential mediators of upstream EGFR signaling during hibernation. Further investigation also revealed a trend toward reduced apoptotic signaling since p-BAD protein levels were greatly suppressed during torpor and caspase nine levels only increased significantly during IA. Thus, the phosphorylation on EGFR sites, that can activate MAPK signals during early and IA, followed by the decrease in EGFR phosphorylation on sites that promote receptor internalization during late torpor, provides a possible upstream mechanism for this MAPK signaling. The subsequent inhibition of apoptosis through phosphorylation cascades and MAPK signaling in hibernator heart seems to agree, if ultimately opposite to data for other tissues (i.e., skeletal muscle) making EGFR phosphorylation a notable addition to the mechanism of cardiac protection during hibernation.

## Supplemental Information

10.7717/peerj.7587/supp-1 Supplemental Information 1 Supplemental Data File. Data values used in study.

10.7717/peerj.7587/supp-2 Supplemental Information 2 Figure 3 pCreb Blot 1. Full Blot for pCreb EC EN LT time points. Box represents quantified bands.

10.7717/peerj.7587/supp-3 Supplemental Information 3 Figure 3 pCreb Blot 2. Full Blot for pCreb EC EA IA time points. Box represents quantified bands.

10.7717/peerj.7587/supp-4 Supplemental Information 4 Figure 3 pELK1 Blot 1. Full Blot for pELK1 EA IA LT time points. Arrow indicates quantified bands.

10.7717/peerj.7587/supp-5 Supplemental Information 5 Figure 3 pELK1 Blot 2. Full Blot for pELK1 EC EN LT time points. Arrow indicates quantified bands.

## Additional Information and Declarations

### Data Availability

The following information was supplied regarding data availability: The raw measurements are available in the Supplemental Files.
