# Supplementary figures and images for: The heart of a hibernator: EGFR and MAPK signaling in cardiac muscle during the hibernation of thirteen-lined ground squirrels, Ictidomys tridecemlineatus
Source: PeerJ. 2019 Sep 5;7:e7587. doi: 10.7717/peerj.7587 (PMC6732209; doi:10.7717/peerj.7587)

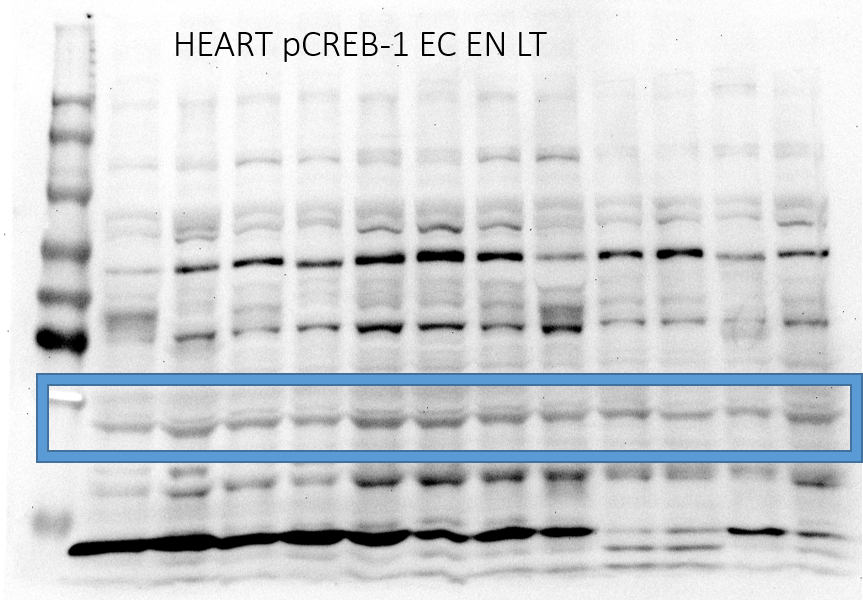

Supplement: Supplemental Information 2 — Full Blot for pCreb EC EN LT time points. Box represents quantified bands. [file peerj-07-7587-s002.png]

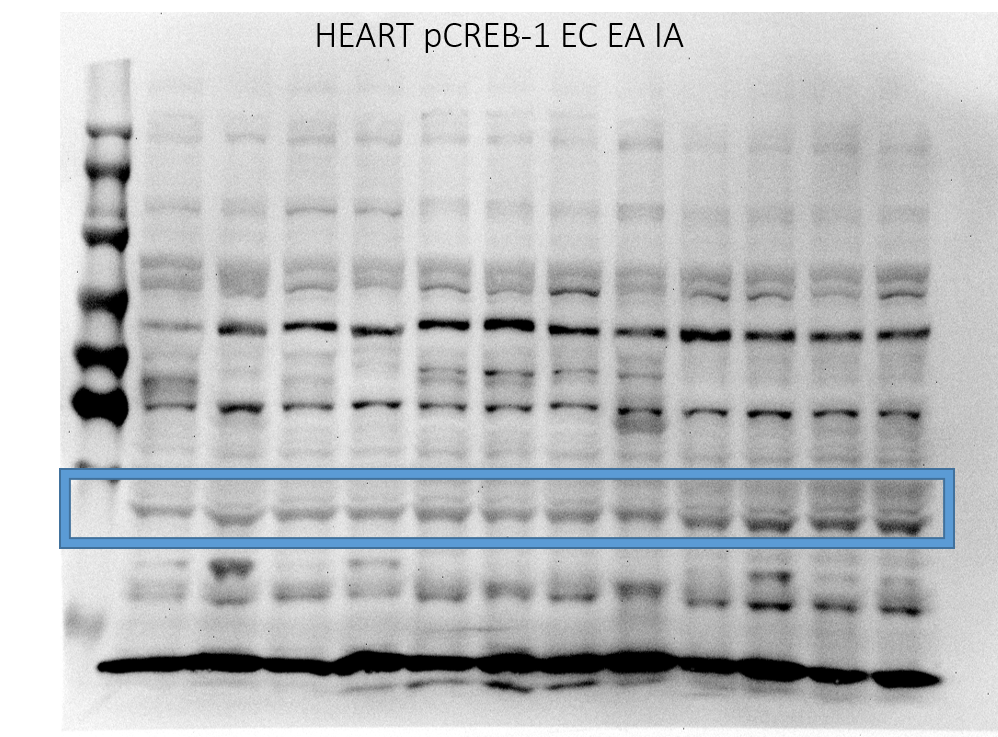

Supplement: Supplemental Information 3 — Full Blot for pCreb EC EA IA time points. Box represents quantified bands. [file peerj-07-7587-s003.png]

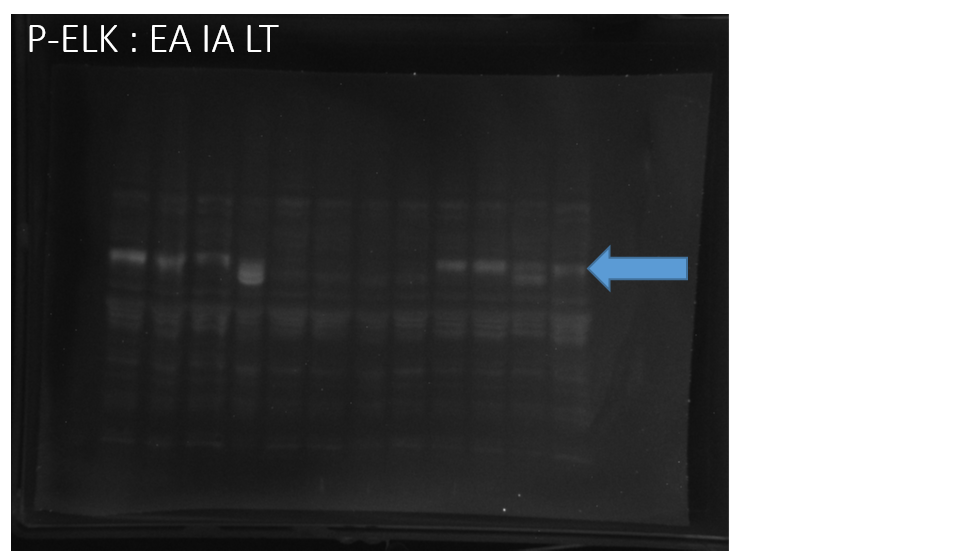

Supplement: Supplemental Information 4 — Full Blot for pELK1 EA IA LT time points. Arrow indicates quantified bands. [file peerj-07-7587-s004.png]

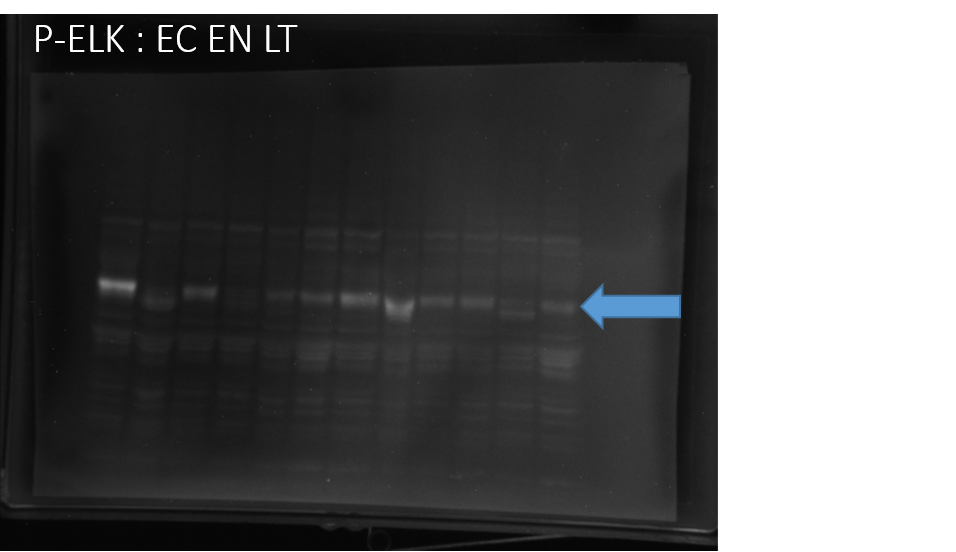

Supplement: Supplemental Information 5 — Full Blot for pELK1 EC EN LT time points. Arrow indicates quantified bands. [file peerj-07-7587-s005.png]
